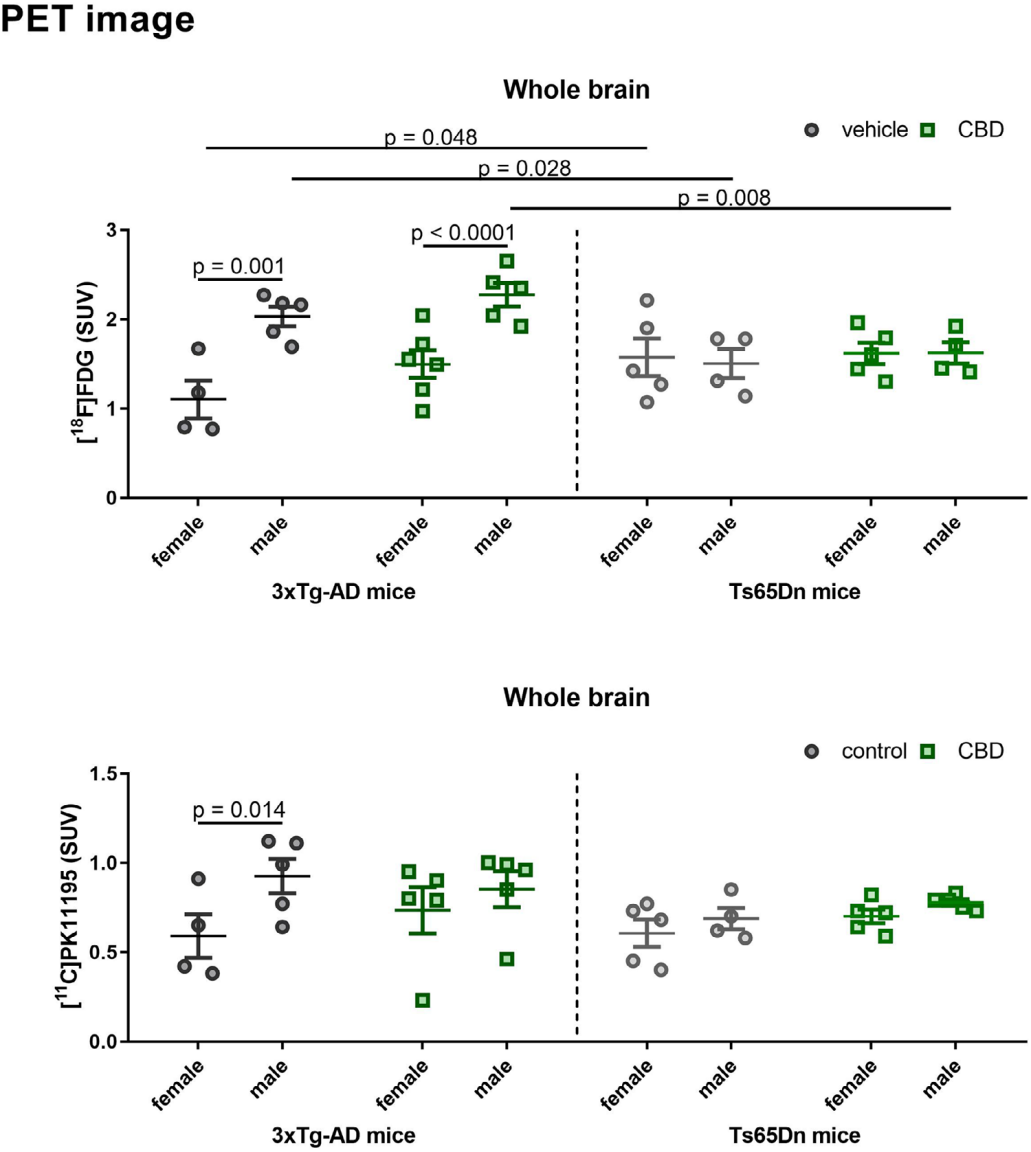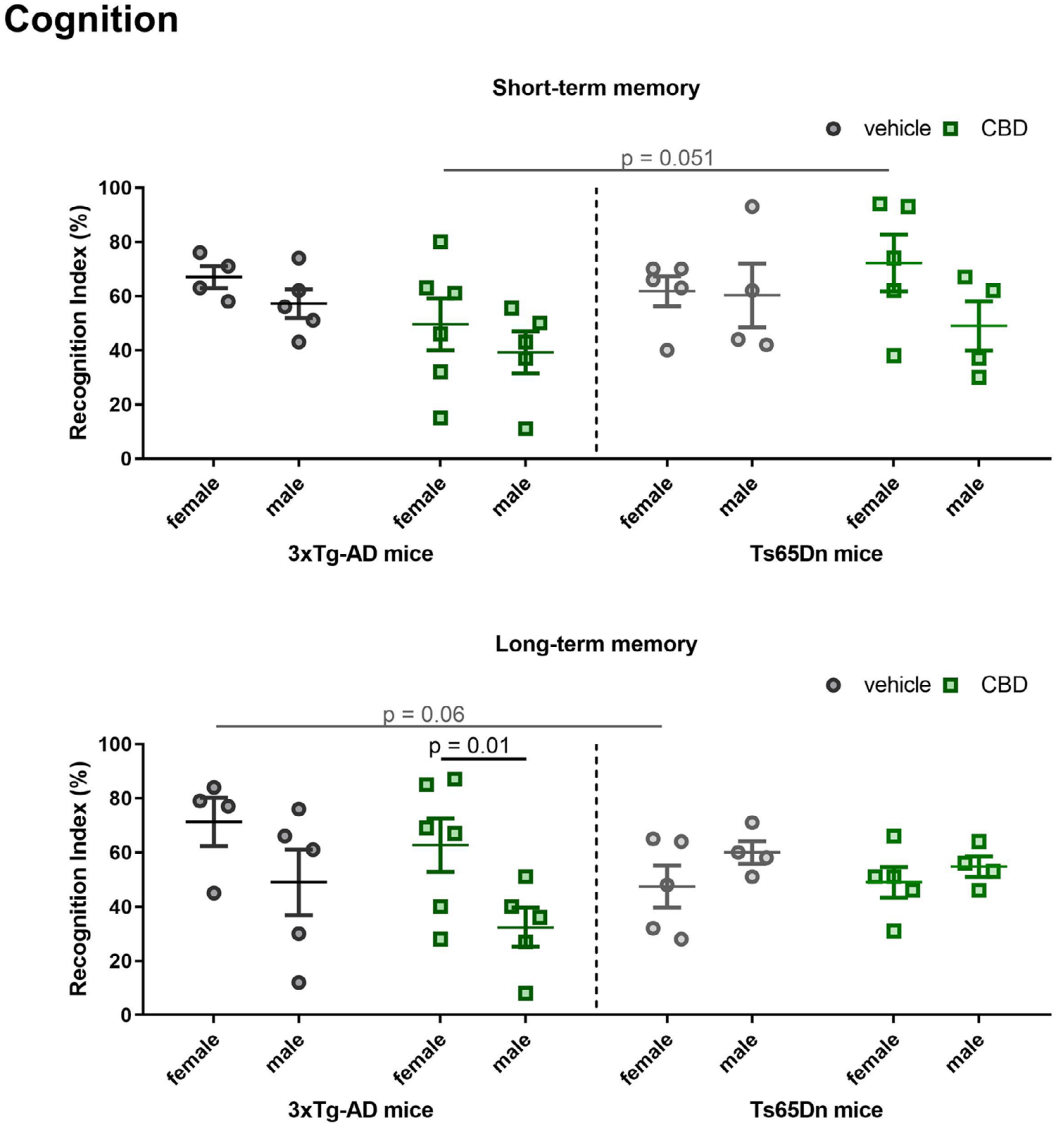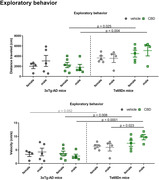# Sex‐ and lineage‐specific effects of cannabidiol in mouse models of Alzheimer’s Disease – a PET scan and behavioral study

**DOI:** 10.1002/alz.090662

**Published:** 2025-01-09

**Authors:** Lidia Emmanuela Wiazowski Spelta, Chiara Maria Righini, Jean Marques Brizola, Daniele de Paula de Paula Faria

**Affiliations:** ^1^ University of São Paulo Medical School, São Paulo, São Paulo Brazil

## Abstract

**Background:**

Emerging preclinical studies show that cannabidiol (CBD) has neuroprotective and anti‐inflammatory effects that may have the potential to improve Alzheimer's disease (AD) therapy. Although much progress has been made in understanding the pathology of AD, its multifactorial nature can't be mimicked in a single preclinical model. In order to improve preclinical results and search for AD better interventions, the aim of this study is to compare the effects of CBD in two AD animal models in a sex‐dependent manner.

**Method:**

Male and female Ts65Dn and 3xTg‐AD mice were used in this study (Ethical approval 1811/2022). At 7 months‐old animals received CBD (20mg/kg, ip) or vehicle (etanol:tween‐80:saline) for 30 days. At 8 months‐old, animal behavior was evaluated in the open field to access exploratory behavior and memory, through the novel object recognition test. Animals were also inject with [^18^F]FDG (5.5MBq, iv) and [^11^C]PK11195 (29.6–7.4MBq, iv) to evaluate neurodegeneration and neuroinflammation by positron emission tomography (PET).

**Result:**

Exploratory behavior had significant influence of sex, animal lineage and treatment. CBD‐treated male and female 3xTg‐AD mice traveled less distance in the open field than the Ts65Dn ones (p=0.025 comparing females; p=0.004 comparing males), and had a smaller velocity (p=0.008 comparing females and p<0,0001 comparing males). Also, in male Ts65Dn mice, the velocity was higher in CBD‐treated than in vehicle‐treated group (p=0.023). Considering memory, there was an effect of sex and CBD in 3xTg‐AD mice, as the long‐term‐memory recognition index was higher in CBD‐treated females than in males (p=0.01). Regarding whole brain PET, [^18^F]FDG uptake was higher in 3xTg‐AD males compared to females, in vehicle‐treated (p=0.001) and CBD‐treated (p<0.0001) groups. Also, in male animals [^18^F]FDG uptake was higher in 3xTg‐AD than in Ts65Dn treated with vehicle or CBD (p=0.028 and p=0.008, respectively). Finally, in vehicle‐females [^18^F]FDG uptake was lower in 3xTg‐AD compared to Ts65Dn mice (p=0.048). Considering [^11^C]PK11195 uptake, the only significant difference was a higher uptake in 3xTg‐AD vehicle‐treated males compared to females (p=0.014).

**Conclusion:**

Our study highlights the importance of studying sex in preclinical research, as well the development of different mouse models for AD research. This would strengthen the translational power of preclinical research.